# Factors related to breast cancer detection mode and time to diagnosis in Alberta, Canada: a population-based retrospective cohort study

**DOI:** 10.1186/s12913-016-1303-z

**Published:** 2016-02-19

**Authors:** Yan Yuan, Maoji Li, Jing Yang, Tracy Elliot, Kelly Dabbs, James A. Dickinson, Stacey Fisher, Marcy Winget

**Affiliations:** School of Public Health, University of Alberta, Edmonton, Alberta T6G 1C9 Canada; Cancer Control Alberta, Alberta Health Services, Edmonton, Alberta T5J 3H1 Canada; Department of Diagnostic Imaging, Foothills Medical Centre, Calgary, Alberta T2N 2T9 Canada; Department of Surgery, University of Alberta, Edmonton, Alberta T6G 1C9 Canada; Family Medicine and Community Health Sciences, University of Calgary, Calgary, Alberta T2N 4N1 Canada; Divison of General Medical Disciplines, Stanford University School of Medicine, Stanford, CA 94305 USA

**Keywords:** Breast cancer diagnosis, Population study, Diagnostic interval, Administrative data

## Abstract

**Background:**

Understanding the factors affecting the mode and timeliness of breast cancer diagnosis is important to optimizing patient experiences and outcomes. The purposes of the study were to identify factors related to the length of the diagnostic interval and assess how they vary by mode of diagnosis: screen or symptom detection.

**Methods:**

All female residents of Alberta diagnosed with first primary breast cancer in years 2004–2010 were identified from the Alberta Cancer Registry. Data were linked to Physician Claims and screening program databases. Screen-detected patients were identified as having a screening mammogram within 6-months prior to diagnosis; remaining patients were considered symptom-detected. Separate quantile regression was conducted for each detection mode to assess the relationship between demographic/clinical and healthcare factors.

**Results:**

Overall, 38 % of the 12,373 breast cancer cases were screen-detected compared to 47 % of the screen-eligible population. Health region of residence was strongly associated with cancer detection mode. The median diagnostic interval for screen and symptom-detected cancers was 19 and 21 days, respectively. The variation by health region, however, was large ranging from an estimated median of 4 to 37 days for screen-detected patients and from 17 to 33 days for symptom-detected patients. Cancer stage was inversely associated with the diagnostic interval for symptom-detected cancers, but not for screen-detected cancers.

**Conclusion:**

Significant variation by health region in both the percentage of women with screen-detected cancer and the length of the diagnostic interval for screen and symptom-detected breast cancers suggests there could be important differences in local breast cancer diagnostic care coordination.

## Background

Breast cancer is the most commonly diagnosed cancer among Canadian women and the second most common cause of their cancer death [1]. Prognosis is best when diagnosed and treated at an early stage; timely diagnosis and early detection are, therefore, critical to optimizing survival. In Canada, early detection is facilitated by guideline recommendation of routine screening for breast cancer with mammography for average risk women aged 50 to 69 every 2 years; also, the Canadian Association of Radiologists recommends that women between 40 and 49 also be screened annually [2, 3]. Similar recommendations and breast cancer screening programs exist in most developed countries, as screening has been shown to reduce mortality by 20 to 30 % [4–6].

Regardless of the mode of detection, timely diagnostic resolution following detection of breast abnormalities is important. Delayed resolution is associated with larger tumors, locally advanced or metastatic cancer, higher recurrence rates, lower 5-year survival rates, and increased patient anxiety [7–9]. Diagnostic delays of three months or more in symptom-detected breast cancer are associated with a 12 % lower 5-year survival compared to those with shorter delays [10]. Delayed resolution of false positive screening mammograms has also been associated with decreased participation in subsequent screening [11]. Given the public health impact of breast cancer and the value of early detection and timely diagnosis, it is important to understand factors related to timely breast cancer diagnosis and the relationship between mode of diagnosis and diagnostic interval to optimize the patient care experience and, ultimately, survival of breast cancer patients.

Here we investigate mode of detection and time to breast cancer diagnosis in Alberta, Canada and report findings on 1) the proportion of screen vs. symptom-detected breast cancers, 2) time to diagnosis by mode of detection and 3) identify patient demographic, clinical and healthcare system factors related to mode of detection and time to diagnosis.

## Methods

### Study population

A retrospective breast cancer cohort of female residents of Alberta, who were diagnosed with a histologically confirmed first-ever primary breast cancer (International Classification of Disease for Oncology (ICD-O) 3rd edition code C50 behaviors 2 and 3 [12]) in years 2004 to 2010, was identified from the Alberta Cancer Registry, a population based cancer registry recognized for data completeness by the North American Association of Central Cancer Registries. All patients were included unless data to calculate outcome measures, as defined below, were missing.

During the study period, the province of Alberta had a population of about 3 to 3.5 million spread over 662,000 km^2^ [13]. Approximately 56 % of the total population live within the two urban regions, 27 % live in small and medium regional cities, and roughly 17 % live in rural and remote areas [14]. Alberta has a publicly-funded provincially operated single-payer health care system, in which all residents have free access to standard medical care. The majority of physicians are remunerated via fee-for-service. Typically women are referred for mammography by their primary care physician (PCP) but screen-eligible patients can self-refer.

### Data sources and variables

The following patient characteristics were obtained from the Alberta Cancer Registry: age, postal code of residence, regional health authority (RHA) of residence, disease stage, histologic grade and date of diagnosis. Disease stage was based on the American Joint Committee on Cancer staging [15]. Histology grade was based on the ICD-O morphology grade that represents the degree of differentiation of the tumor.

The postal codes of patients were used to obtain neighbourhood-level variables developed by Statistics Canada. Specifically, neighbourhood income level (in quintiles, QAIPPE) and an urban/rural variable (CSIZEMIZ) were obtained by linking patient postal codes to the 2006 Canadian Census Data [16]. The latter variable is defined by categorizing communities with populations less than 10,000 that have no influence from larger cities as rural and all other communities as urban.

International Classification of Disease, Tenth Revision, Clinical Modification [ICD-10-CM] diagnosis codes from three provincial administrative health care databases were used to calculate the Charlson Comorbidity Index: 1) the Ambulatory Care Classification System, all outpatient visits to hospitals in the province; 2) the Discharge Abstract Database, all inpatient hospital admissions in the province, and 3) the Physician Claims Database, all fee-for-service physician visits. All relevant codes in the period 30 months prior to breast cancer diagnosis were used to calculate the modified Charlson Comorbidity Index, [17, 18]. Codes for primary or metastatic cancer were excluded from calculations.

The Physician Claims Database was used to identify visits to a PCP using the ‘provider type’ code to calculate the Usual Provider Continuity (UPC) score. The UPC is calculated by dividing the number of visits to the PCP the patient has seen the most by the total number of all PCP visits a patient has had in a given time period [19, 20]. In order to capture typical PCP utilization, all PCP visits from 6 to 30 months prior to cancer diagnosis were included in the UPC calculation and a minimum of three PCP visits was required to calculate the UPC. Patients with less than three PCP visits in the period were classified as having “minimum PCP visits”.

The databases and datasets used in the study are not publicly available. The Alberta Cancer Registry data were made available upon ethics approval. The provincial administrative databases are governed by Alberta Health Services (AHS) via permission from the provincial ministry, Alberta Health (AH). AHS provided the provincial administrative data required for the study after reviewing the study protocol, receiving a signed confidentiality agreement (from MW) and receiving proof of ethics approval. Ethics approval for the study was obtained from the University of Alberta Health Research Ethics Board.

### Outcome measures

The outcome measures are percent screen-detected cancers and the length of the diagnostic interval. All breast cancer related diagnostic procedures, including screening/diagnostic mammograms, breast ultrasound and breast biopsies, were identified from the Physician Claims Database and the Screen Test Database. These two databases are complementary, capturing all breast cancer related screening and diagnostic procedures in the province from fee-for service and salaried radiologists, respectively. All breast-related procedures within a validated look-back period [21] from the date of diagnosis in the Alberta Cancer Registry were obtained.

**Fig. 1:**
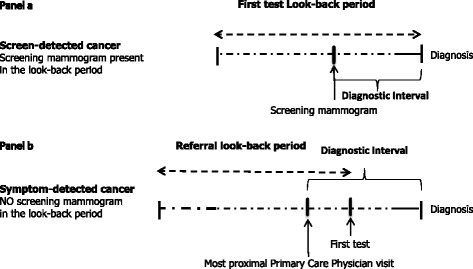
An illustration of the diagnostic interval for screen and symptom detected breast cancers. For the screen-detected cancers (panel **a**), the diagnostic interval is the time between the date of diagnosis and the date of the screening mammogram, a maximum of 6 months. For the symptom-detected cancers (panel **b**), the diagnostic interval is the time between the date of diagnosis and the date of the most proximal GP visit prior to the first diagnostic test, a maximum of 12 months

Breast cancer was defined as screen-detected if the patient had a screening mammogram within the look-back period. The length of the diagnostic interval was defined as the time from the date of the screening mammogram to the date of breast cancer diagnosis, typically the first positive percutaneous or surgical biopsy date. The remaining cancers were defined as symptom-detected. The earliest breast cancer related diagnostic procedure in the look-back period, usually a diagnostic mammogram, was defined as the first relevant diagnostic test. The most proximal visit to a PCP within 6-months prior to the first relevant test was defined as the start of the diagnostic interval for symptom-detected patients, because in most cases a diagnostic test can only be conducted if a referral is made by a PCP. The diagnostic interval for symptom-detected patients, therefore, is defined as the time interval from the date of the PCP visit to the date of cancer diagnosis. Figure 1 summarizes the diagnostic interval by detection method as described above.

Data were linked using the unique provincial healthcare identification number which was anonymized for data analysis. Quality assurance and cross checks were performed on data sets during and after data linkage to ensure accuracy and completeness. Ethical approval was obtained from the Human Research Ethics Board at the University of Alberta before conducting the study.

### Statistical analyses

Demographic, clinical and healthcare system factors were assessed for their univariate associations with each of the outcome measures. Chi-square tests were calculated to assess associations with percent screen-detected breast cancers and Kruskal-Wallis tests were calculated to assess associations with the length of the diagnostic interval. The demographic factors evaluated were age and neighbourhood income quintile. Patients were grouped as follows: 39 and under, 40–49, 50–69, 70–74, and 75 and over. The age groupings were based on screening guidelines and clinical practice in Alberta: 50–69 is the screen-eligible age group during the study period [22]; women aged 70–74 are included in the most recent breast screening guideline [23]; and women aged 40–49 years old can be referred for breast cancer screening in practice. Clinical factors included cancer stage at diagnosis, histology grade, and Charlson comorbidity index. Healthcare system factors included time period of cancer diagnosis, Usual Provider Continuity score (UPC), regional health authority (RHA), and urban/rural residence. The cut point for the time period variable was chosen based on the observed trend of percent screen-detected cancers over the years. Time period 1 includes cancers diagnosed in years 2004 to 2006 and time period 2 includes cancers diagnosed in years 2007 to 2010.

Multivariable logistic regression was used to examine the association of factors on the likelihood of cancer being screen-detected. The most parsimonious model was identified and presented as the final model. The interaction of time period by RHA was hypothesized and tested; the interaction was statistically significant and kept in the final model. For the diagnostic interval outcome, multivariable quantile regression models were fitted to estimate the effects of individual factors and the interaction effects between RHA and time period for screen and symptom detected patients separately. Briefly, quantile regression is similar to linear regression; it differs by modeling the median or specific quantiles defined by the user rather than the mean. For non-symmetric distributions such as time intervals, quantile regression is more appropriate than linear regression. The estimates from quantile regression are the difference in diagnostic interval (days) associated with each factor, making it simple to understand. Quantile regression models were run for the median and the 90^th^ percentile; the 90^th^ percentile was intended to represent diagnostic delay. All analyses were conducted using SAS 9.4 (SAS Institute, Cary NC).

## Results

### Mode of diagnosis

There were 12,813 first-ever female breast cancers diagnosed in Alberta residents in years 2004 to 2010; 440 (3.4 %) were excluded from the study because of missing data that prevented the assignment of detection mode. Table 1 gives the descriptive statistics of the demographic, clinical, and healthcare system factors of the breast cancer patient cohort by detection mode. About 38 % of the cancer cases were screen-detected overall. Roughly 50 % of patients were in the screen-eligible age group (50 to 69 years of age), of whom 47 % were screen-detected. In the whole cohort, the majority of screen-detected patients, 72 %, had in situ or stage I cancer compared to only 39 % in the symptom-detected patients (*P* < 0.001). Similarly, screen-detected cancers had a lower histological grade than the symptom-detected cancers; 25 % of screen-detected tumors were low grade compared to 16 % of symptom detected (*P* < 0.001). About 57 % of screen-detected in situ cancers, however, were high grade (nuclear) compared to 50 % of those symptom-detected (*P* = 0.01, data not shown). Similar contrasts by detection mode were found on stage and histology grade among screen-eligible patients. Patients with a high UPC score were more likely to be screen-detected than those with a low score or those with minimum PCP visits, 42, 37, and 31 %, respectively (*P* < 0.0001). Rural patients were more likely to be symptom-detected than urban patients: 67 and 60 %, respectively (*P* < 0.001).

**Table 1:** Relationship between demographic, clinical, and healthcare factors and breast cancer detection mode

Factor	Total	Screen detected		Symptom detected	
	N (12373)	N (4747)	%^a^ (38.4)	N (7626)	%^a^ (61.6)
Demographic factors					
Age*					
39-	661	0	(0)	661	(100)
40–49	2660	838	(31.5)	1822	(68.5)
50–69	6046	2857	(47.3)	3189	(52.7)
70–74	1056	487	(46.1)	569	(53.9)
75+	1950	565	(29.0)	1385	(71.0)
Neighborhood Income Quintile*					
> 80 %	2694	1121	(41.6)	1573	(58.4)
60 % ~ 80 %	2388	922	(38.6)	1466	(61.4)
40–60 %	2547	967	(38.0)	1580	(62.0)
20 % ~ 40 %	2443	911	(37.3)	1532	(62.7)
< 20 %	2247	809	(36.0)	1438	(64.0)
Missing	54	17	(31.5)	37	(68.5)
Clinical factors					
Stage*					
In Situ	1588	967	(60.9)	621	(39.1)
I	4803	2440	(50.8)	2363	(49.2)
II	3821	1017	(26.6)	2804	(73.4)
III	1489	230	(15.4)	1259	(84.6)
IV	405	43	(10.6)	362	(89.4)
Missing	267	50	(18.7)	217	(81.3)
Histological Grade*					
Well differentiated	2359	1165	(49.4)	1194	(50.6)
Moderately differentiated	4976	1977	(39.7)	2999	(60.3)
Poorly or undifferentiated	4408	1331	(30.2)	3077	(69.8)
Unknown/Not stated/Not applicable	630	274	(43.5)	356	(56.5)
Charlson Comorbidity Index*					
0	8961	3507	(39.1)	5454	(60.9)
1	2348	912	(38.8)	1436	(61.2)
> =2	1064	328	(30.8)	736	(69.2)
Healthcare factors					
Time Period*					
2004–2006	5004	1732	(34.6)	3272	(65.4)
2007–2010	7369	3015	(40.9)	4354	(59.1)
Usual Provider Continuity*					
High continuity (>0.75)	5037	2104	(41.8)	2933	(58.2)
Low continuity (<=0.75)	5731	2139	(37.3)	3592	(62.7)
Minimum doctor visits	1605	504	(31.4)	1101	(68.6)
Community Size and Metropolitan Influence Zone*					
Urban	9741	3872	(39.7)	5869	(60.3)
Rural	2632	875	(33.2)	1757	(66.8)

^a^Percentages are row percents. *P-value < 0.001

**Fig. 2:**
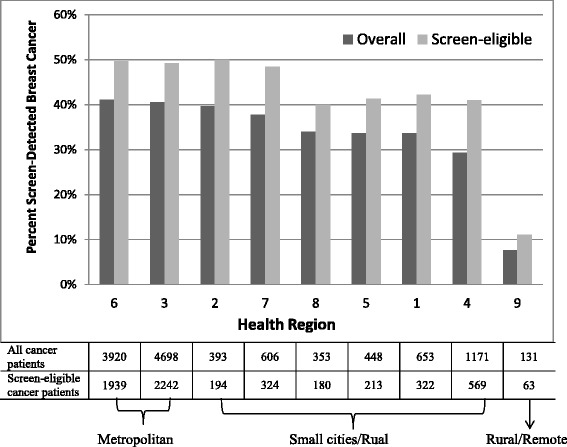
Percent of screen-detected breast cancer by health region (RHA). The black histograms represent the percent in the entire patient cohort and the grey histograms represent the percent in the screen-eligible patients, i.e. those aged 50 to 69 years. RHAs are ordered according to the percent screen-detection from high to low in the entire patient population

The variation in percent screen-detected cancers by RHA for all patients and screen-eligible patients only is shown in Fig. 2. Approximately 50 % of screen-eligible women were screen-detected in four RHAs, 40 % in four RHAs and only 10 % in one RHA.

**Fig. 3:**
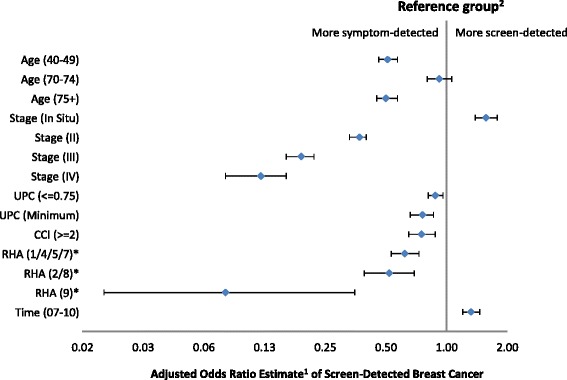
The adjusted odds ratio of screen-detected female breast cancer diagnosed in Alberta, Canada (2004–2010).^1^ Adjusted for all the variables shown plus the interaction terms of RHA by Time Period; ^2^The reference group is: age group 50–69, cancer stage 1, usual provider continuity > 0.75, Charlson comorbidity index 0 or 1, RHA 3/6, and time period 2004–2006.*RHAs that have similar estimated odds were grouped together. The reference RHAs are the metropolitan regions, 3&6

Figure 3 shows the forest plot of the adjusted odds ratios from the multivariable logistic regression model in which the outcome is screen-detected cancer. RHAs that had similar estimated odds were grouped together. Patients outside of the screening-eligible age group were less likely to be screen-detected except for age group 70–74 (OR: 0.93, 95 % CI: 0.80–1.06). The likelihood of being screen-detected increased with decreasing disease stage. Compared to stage I cancer, the estimated odds ratio of being screen-detected was 1.57, 0.37, 0.19 and 0.12 for in situ, stages II, III, and IV cancers, respectively (*P* < 0.001). Having a comorbidity index greater than or equal to 2 was associated with a decreased odds of being screen-detected comparing to those with a comorbidity index less than 2 (OR: 0.73, 95 % CI: 0.65–0.88).

**Fig. 4:**
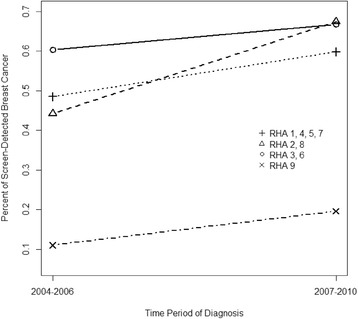
An illustration of the effect modification on percent screening-detection. The percent screening detected female breast cancers in different RHAs was modified by the time period in women diagnosed in Alberta, Canada in years 2004–2010

Figure 4 shows significant interaction between RHA and time period. The probability of screen-detected cancer increased in all regions from 2004–2006 to 2007–2010, however, it increased to a greater extent in RHAs 2 and 8 than other RHAs (*P* < 0.001).

### Diagnostic interval

Table 2 shows the unadjusted time to diagnosis by detection method. Three hundred forty six symptom-detected patients (4.5 % of symptom-detected patients) were excluded from the analysis because they did not have a visit with a PCP within 6 months prior to their first diagnostic test. The median (90^th^ percentile) time to diagnosis was 19 (70) and 21 (92) days for screen- and symptom-detected breast cancers, respectively. Cancer stage was inversely related to the diagnostic interval in symptom-detected women: as cancer stage increased from in situ to stage IV, the median diagnostic interval decreased from 49 to 13 days. The relationship was much attenuated in the screen-detected group, however; in situ cancers had a median time of 30 days while all invasive cancers (stage I through IV) had a median of about 16 days. There was also significant variation in time to diagnosis by health region: the median diagnostic interval ranged from 7 to 42 days in the screen-detected group and from 13 to 33 days in the symptom-detected group.

**Table 2:** Median and 90^th^ percentile in days of the diagnostic interval by detection mode

Factor	Screen-detected (*n* = 4747)			Symptom-detected (*n* = 7280)		
	Median	90 %	*P*-value	Median	90 %	*P*-value
Overall	19	70		21	92	
Demographic factors						
Age						
39-	NA	NA	0.10	21	80	<0.001
40–49	21	91		21	98	
50–69	19	69		21	100	
70–74	19	66		23	106	
75+	18	60		20	70	
Neighborhood Income Quintile						
> 80 %	17	77	0.046	21	102	0.49
60 % ~ 80 %	20	70		21	93	
40–60 %	19	66		21	98	
20 % ~ 40 %	19	70		22	88	
< 20 %	22	68		21	83	
Missing	37	130		26.5	75	
CLINICAL FACTORS						
Stage						
In Situ	30	87	<0.001	49	156	<0.001
I	15	57.5		25	106	
II	16	71		19	66	
III	15	123.5		17	71	
IV	16	83		13	65	
Missing	38	133.5		19.5	97	
Histological Grade						
Well differentiated	17	62	0.008	28	112	<0.001
Moderately differentiated	18	68		21	93	
Poorly or undifferentiated	20	73		19	74	
Unknown/ Not stated/ Not applicable	34	100		35	135.5	
Charlson Comorbidity Index						
0	19	70	0.067	21	98	0.004
1	21	75		20	81	
≥ 2	19	63		20	86	
Healthcare factors						
Regional Health Authority						
1	29.5	83.5	<0.001	26	106	<0.001
2	41.5	110		33	128	
3	25	73		25	98	
4	29	70		22	84	
5	19	62		19	100	
6	7	54		17	87	
7	19	69		16	69	
8	29.5	181		13	86	
9	29.5	57.5		14	58	
Time Period						
2004–2006	18	67	0.024	21	87	0.72
2007–2010	20	73		21	97	
Usual Provider Continuity						
High continuity (>0.75)	19	67	0.48	22	91	<0.001
Low continuity (<=0.75)	20	74		22	99	
Minimum doctor visits	18	70		18	63	
Community Size and Metropolitan Influence Zone						
Urban	17	69	<0.001	21	93	0.01
Rural	26	72		20	88	

Table 3 shows the results of the quantile regression models for the median and the 90 % quantile for the two detection methods, based on a simplified model that did not include any interaction term.

**Table 3:** Quantile regression estimates of the median and 90^th^ percentile of diagnostic interval by detection mode. The estimate associated with each category is the difference in days when compared to the reference variable category

Factor	Screen-detected (*n* = 4747)				Symptom-detected (*n* = 7280)			
	Quantile 50 %		Quantile 90 %		Quantile 50 %		Quantile 90 %	
	Days	*P*-value	Days	*P*-value	Days	*P*-value	Days	*P*-value
Intercept								
	4.0	**	38.7	**	20.4	**	108.5	**
Demographic factors								
Age								
50–69 (Ref.)								
39-	NA	NA	NA	NA	−1.0	0.29	−9.7	0.05
40–49	0	1.00	14	*	−0.8	0.19	1.6	0.72
70–74	0	1.00	4.9	0.26	1.2	0.42	−1.2	0.88
75+	1.0	0.19	−4.9	0.19	−1.4	0.07	−19.7	**
Neighborhood income quintile								
> 80 % (Ref.)								
60 % ~ 80 %	0	1.00	−3.3	0.52	1.6	0.06	−7.4	0.16
40–60 %	1.0	0.17	−6.9	0.15	0.6	0.44	−3.9	0.48
20 % ~ 40 %	2.0	**	−2.9	0.54	1.8	*	−7.4	0.20
< 20 %	2.0	**	−8.4	0.11	1.4	0.11	−9.4	0.10
Missing	16.0	0.19	63.0	0.52	5.4	0.42	−11.7	0.78
Clinical factors								
Stage								
I (Ref.)								
In Situ	11.0	**	28.0	**	24.8	**	49.2	**
II	0.0	1.00	9.3	*	−6.4	**	−36.7	**
III	0.0	1.00	60.8	**	−7.0	**	−29.8	**
IV	−3.0	0.27	16.0	0.42	−12.2	**	−37.7	**
Missing	16.0	**	37.4	0.16	−5.6	*	−1.5	0.87
Charlson comorbidity index								
0/1 (Ref.)								
> =2	1.0	0.22	−3.1	0.56	−0.8	0.50	7.3	0.11
Healthcare factors								
Regional Health Authority								
6 (Ref.)								
1	20.0	**	37.5	**	7.8	**	16.3	0.07
2	30.0	**	50.2	**	12.6	**	30.5	**
3	16.0	**	18.9	**	7.4	**	5.7	0.20
4	19.0	**	19.3	*	6.0	**	−2.5	0.71
5	10.0	**	21.4	0.12	2.8	0.17	4.7	0.70
7	6.0	*	9.6	0.22	1.2	0.47	−21.5	*
8	22.0	**	118.8	**	−3.4	0.06	−9.3	0.54
9	21.0	*	6.4	0.97	−3.0	0.27	−17.7	*
Time Period								
2004–2006 (Ref.)								
2007–2010	1.0	*	5.9	0.06	0.2	0.70	6.1	0.06
Usual Provider Continuity								
High continuity (>0.75, Ref.)								
Low continuity (<=0.75)	−0.0	1.00	5.4	0.12	0.4	0.50	2.9	0.46
Minimum doctor visits	−1.0	0.26	−2.77	0.62	−2.0	*	−19.2	**
Community Size and Metropolitan Influence Zone								
Urban (Ref.)								
Rural	3.0	*	−2.3	0.66	−1.2	0.20	1.7	0.75

**P-value < 0.005 *P-value < 0.05

**Fig. 5:**
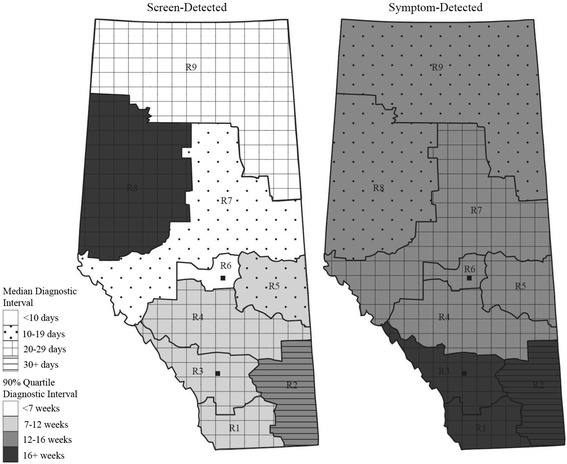
Maps of Alberta displaying regional variation in diagnostic interval by detection mode. The model based median and 90^th^ percentile estimates of the diagnostic interval are for urban patients with stage I cancer, in age group 50–69, and diagnosed in years 2004–2006. The map legends R1 – R9 represent RHA1 – RHA9. The two solid squares in R3 and R6 indicate the location of the two metropolitan areas

RHA and cancer stage had the strongest associations with the time to diagnosis for both screen- and symptom-detected cancers, though the associations differed by detection mode. The estimated median time to diagnosis ranged from 4 to 37 days depending on the RHA in the screen-detected cancers (*P* < 0.005) and from 17 to 33 days in the symptom-detected cancers (*P* < 0.005), holding other factors at the reference levels. The dissimilar patterns of the adjusted median and 90^th^ percentile diagnostic intervals depicted in Fig. 5 illustrate the extent of regional variation. A longer median (90^th^ percentile) in a given RHA for a given detection mode does not necessarily translate into a longer median (90^th^ percentile) for the other detection mode in the same RHA.

Similarly, cancer stage showed a different pattern of variation in the screen- and symptom-detected groups. In symptom-detected cancers, the estimated median time to diagnosis decreased monotonically with increasing stage (45, 20, 14, 13 and 8 days for in situ, stage I, II, II, and IV, respectively, *P* < 0.005); the estimated median time in screen-detected cancers also decreased with increasing stage but had a much smaller range (15, 4, 4, 4 and 1 days for in situ, stage I, II, II, and IV, respectively, *P* < 0.005), holding other factors at the reference levels.

**Fig. 6:**
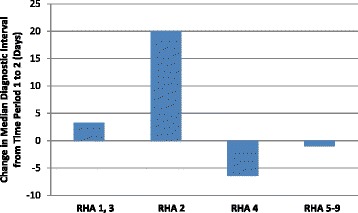
An illustration of the effect modification on the median diagnosis interval in symptom-detected cancers. The length of diagnostic interval in different RHAs was modified by time period in women diagnosed in Alberta, Canada in years 2004–2010. In some regions the median diagnostic interval increased whereas in others it decreased over time. Note that RHAs are grouped differently in Fig. 6 compared to Fig. 4, because different outcomes are illustrated in the two figures. There are different patterns by RHA for the two outcomes

For symptom-detected patients, the effect of RHA on the length of the diagnostic interval was modified by the time period.^1^ Figure 6 illustrates the effect modification of time period on RHA observed in the median time to diagnosis. From 2004–2006 to 2007–2010, the median diagnostic interval increased dramatically by 18 days in RHA 2 (*P* < 0.001), increased by 3 days in RHAs 1, 3 and 8 (*P* < 0.001), decreased by about 6 days in RHA 4 (*P* < 0.001), and remained largely the same in RHAs 5, 6, 7, and 9. Overall, the median diagnostic intervals were less than a month for all RHAs except RHA 2 in 2007–2010, where the median diagnostic interval was 41 days.

## Discussion

In Alberta Canada, a province with a free, long-standing, organized screening program, about 38 % of cancers were screen-detected; among the age-eligible population it was 47 %. In our study, those aged 70–74 were as likely as those aged 50–69 to have screen-detected cancer. The median time to diagnosis was similar for screen- and symptom-detected breast cancers at 19 and 21 days, respectively. At the 90^th^ percentile, however, the time to diagnosis was 70 days in screen-detected breast cancers compared to 92 days in symptom-detected breast cancers. Region of residence and cancer stage had the strongest associations with both detection mode and time to diagnosis.

The percent of screen-detected cancers varied from about 10 to 50 % by RHA, however, one RHA was responsible for most of the variation as eight of the nine RHAs ranged from 40–50 % of age-eligible women being screen-detected. The RHA with only 10 % screen-detected breast cancers is a geographically large rural/remote region in the northern-most part of the province; approximately 20 % of the population is Aboriginal [24]. Some of the remote communities only have access to screening mammography through a mobile unit via an ice road during winter months. Other studies have reported similar challenges in the screening of breast cancer among vulnerable populations in such remote regions [25]. The screening program has now developed a special program to encourage screening (for all screeenable cancers) in this region.

As expected, screen-detected cancers were much more likely to be early stage tumours (in situ or stage I) than symptom-detected cancers. In situ cancers accounted for 20 % of screen-detected cancers and 8 % of symptomatic cancers, similar to findings in other jurisdictions [26]. Compared to symptom-detected cancers, screen-detected cancers also tended to have a lower histological grade. Although early detection through screening has long-term survival advantage [4–6, 27], it also results in “over” detection leading to over treatment in a proportion of women [26, 28]. From the current study we are not able to assess this fraction. More research is needed to assess optimal histopathologic diagnosis [29] and treatment for in situ cancers to minimize patient harm and resource waste caused by over treatment.

Cancer patients with a co-morbidity index two or above and those with a low UPC index were less likely to be detected by screening. These findings are consistent with the literature, suggesting that dealing with chronic diseases may divert patients’ and health care providers’ attention from preventive cancer care services [30]. Further, if women have other diseases that are likely to be fatal within a few years, screening may not be appropriate. Patients ‘attached’ to a primary care physician are more likely to receive preventive care [31–33].

The patterns of variation in time to diagnosis differed by RHA and stage for each mode of diagnosis. We hypothesize that the rural regions that had the most extreme diagnostic intervals or diagnostic interval increases over time may suffer from lack of specific resources, such as radiologists or pathologists, as shortage of physician and other healthcare resources is associated with diagnostic delay [33–35]. Time to diagnosis also differed between the two metropolitan areas (RHA 3 and 6) with a larger difference in screen-detected cancers. This is likely explained by a program in RHA 6 that specifies the radiologist’s role in arranging follow-up tests on the same day for abnormal mammogram results. It is hypothesized that communication and referral problems could be responsible for variations in diagnostic intervals in urban and rural centers [36–38]. Closer inspection of communication, referral patterns and healthcare resources is needed to identify ways to minimize regional variation in timeliness of diagnostic care.

Higher stage cancers that were symptom-detected were diagnosed more quickly than those with a lower stage. This is consistent with other studies that have found shorter diagnostic intervals for the most symptomatic cases [39, 40]. Upon further inspection we found that most of the variation in time to diagnosis of symptom-detected cancers by cancer stage was due to a longer time from the PCP visit to the first test for low stage cancers than high stage cancers (data not shown), consistent with clinical practice to prioritize diagnostic tests based on patient symptoms. Interestingly, the inverse relationship between time and cancer stage was much less evident for screen-detected cancers, as invasive cancers of all stages had a median diagnostic interval of approximately 16 days. We hypothesize that the procedures and communication between providers following screen-detection of breast abnormalities are more established and coordinated compared to that for symptom-detected breast abnormalities, and since mammography does not clearly differentiate stage, all are treated alike until biopsy results are available. Clear provider communication of test results, and physician documentation of the follow-up plan have been identified as important factors that facilitate patient receipt of follow-up care [36, 11, 41].

We found that 47 % of screen-eligible women by age were screen-detected, which is a little lower than the 55 % reported in West Midlands, UK [42]. In 2009–10, the overall screening rate in Alberta was about 57.3 % which is lower than the target 70 % that the Canadian programs set in 2006 [43] but higher than the Canadian average of about 52 % [44]. Although screening uptake is not optimal, there is a clear and significant stage shift towards earlier stage in the screen-detected cancers compared to the symptom-detected ones observed in the current study, suggesting that the screening program in Alberta is effective. The large regional variation in both percent screen-detected and time to diagnosis, however, suggests there is significant room for improvement.

The major strength of the study is that we studied a population-based incident case cohort that included all women with a first-ever breast cancer diagnosis over a 6-year period. Thus, survivor bias is minimal in our study. It also allows for the assessment of changes over time. The major limitations of this study are: 1) lack of patient level information that may have explained some of the variation in percent screen-detected cancer based on patient choice due to education, screening awareness and different values placed on screening; 2) lack of detailed clinical information that could affect time to diagnosis, such as breast density, which may explain the shorter diagnostic interval at 90th percentile for older patients; and 3) lack of registry data on molecular biomarkers, e.g. ER, PR and triple negative, which is important to understand the implications of delay in diagnosis for the more aggressive phenotypes. Additionally, issues related to access to healthcare services such as access to a PCP, screening mammography, biopsy and pathologist were not investigated due to the limitation of the data sources, however, the RHA variable served as a proxy for the access factors to some extent.

## Conclusions

Population-based studies are important for assessing healthcare efficiency and identifying disparities within the typical clinical practice environment. The significant variation in mode of detection and time to diagnosis across RHAs found in the current study suggests there are important differences in local coordination of breast cancer diagnosis. Similar regional variation in healthcare has been reported in several other jurisdictions, and for other diseases, indicating a need for routine monitoring within and/or across provinces (insurance providers) [45–48]. The public reporting efforts that have begun in the United Kingdom, Canada, and elsewhere [49–51] are good starting points for identifying variation but local insurers and/or healthcare providers must take it upon themselves to properly identify and address root causes to healthcare variation through programming and monitoring in order to properly address them.

## Abbreviations

ICD-O: International Classification of Disease for Oncology
PCP: primary care physician
RHA: regional health authority
UPC: usual provider continuity score

## Footnotes

1 In the analysis of effect modification, health regions have been grouped according to their median diagnostic interval estimates and their changes over time, in order to make Fig. 6 less busy while keeping the important findings intact.
